# Glucosyl Hesperidin Supplementation Prevents Tubulointerstitial Fibrosis and Immune Activation in Diabetic Nephropathy in Mice

**DOI:** 10.3390/nu17030383

**Published:** 2025-01-21

**Authors:** Kotaro Hashimoto, Yuki Yoshida, Mion Kamesawa, Nao Yazawa, Hikaru Tominaga, Rahmawati Aisyah, Siyi Chen, Chanikan Bumrungkit, Seiji Kawamoto, Thanutchaporn Kumrungsee, Noriyuki Yanaka

**Affiliations:** Graduate School of Integrated Sciences for Life, Hiroshima University, Hiroshima 739-8528, Japanskawa@hiroshima-u.ac.jp (S.K.); kumrung@hiroshima-u.ac.jp (T.K.)

**Keywords:** diabetic nephropathy, glucosyl hesperidin, streptozotocin, tubulointerstitial fibrosis, kidney, DNA microarray analysis

## Abstract

Background: Diabetic nephropathy (DN) is a serious condition that can result in end-stage renal failure. Recent evidence has focused on the dietary effects of polyphenols on blood glucose levels and the complications of diabetes. Objectives: In this study, we investigated the protective effect of glucosyl hesperidin (G-Hes), composed of glucose and hesperidin, against streptozotocin (STZ)-induced nephropathy in mice. Methods: We used an STZ-induced diabetic mouse model to investigate the preventive effect of G-Hes on renal pathology. After G-Hes supplementation for 4 weeks, we investigated the renal gene expression profiles using DNA microarray analysis and renal histology to examine the underlying molecular mechanism. Results: G-Hes suppressed the increase in kidney weight without any change in the blood glucose levels. This study identified 511 genes whose expression levels were substantially increased during DN development but were downregulated by G-Hes supplementation. G-Hes prevented mRNA expression associated with renal tubule injury, fibrosis, and immune responses. Notably, G-Hes supplementation considerably decreased the complement component C3 at the mRNA and protein levels in the glomeruli and ameliorated glomerular and mesangial matrix expansion in diabetic nephropathy. Conclusions: G-Hes supplementation is useful in preventing tubulointerstitial fibrosis and inflammation in a mouse model of DN, without exhibiting a hypoglycemic effect.

## 1. Introduction

Diabetic nephropathy (DN), the most common complication of diabetes, often results in end-stage renal failure. Presently, DN affects a considerable number of individuals with diabetes worldwide and accounts for approximately one-third of patients with chronic kidney disease [1]. Several studies have indicated that innate immunity and tubulointerstitial fibrosis are crucial for DN onset and progression [2,3,4,5]. However, DN pathophysiology has not been completely elucidated due to its complications. Preventing DN progression to end-stage renal failure has not been established, although patients with DN are currently clinically treated based on blood glucose and pressure level control. Therefore, new strategies are required to prevent DN [6,7].

Recent studies have revealed that plant phytochemicals prevent DN development. Polyphenols have been widely investigated for their biological activities against DN as antioxidants and anti-inflammatory factors. Furthermore, dietary polyphenols, such as resveratrol and quercetin, have been previously reported to exert potent antioxidant and anti-inflammatory effects by targeting NF-E2-related factor 2 (Nrf2), high mobility group box 1 (HMGB1), nuclear factor-kappa B (NF-κB), and NLR family pyrin domain-containing protein 3 (NLRP3), suggesting that polyphenol supplementation can prevent and improve renal disease progression such as DN [8,9,10,11,12].

Hesperidin is a flavanone abundant in citrus peels and exhibits numerous effects, such as anti-inflammatory, antilipemic, and antihypertensive effects, in preventing numerous diseases [13,14,15]. Particularly, several researchers have examined its anti-inflammatory and antioxidant activities as potential preventive strategies for inflammation-related diseases [16]. Despite its dietary effects, its low water solubility has resulted in challenges in its effective utilization during food product processing. Glucosyl hesperidin (G-Hes) was synthesized via an enzymatic reaction with cyclodextrin glucanotransferase to improve its water solubility [17]. Due to its extremely high water solubility, G-Hes exhibits more than 3-fold higher bioavailability than hesperidin, which may be related to its effective physiological functions [18]. Previously, we demonstrated that G-Hes supplementation markedly suppressed inflammation in adenine-induced kidney injury [19].

In this study, we investigated the protective effect of G-Hes against streptozotocin (STZ)-induced nephropathy in mice and analyzed the renal gene expression profiles. Additionally, we focused on renal complement proteins and mesangial hypertrophy to show the renoprotective effect of G-Hes independent of its hypoglycemic effects. This study emphasizes the efficacy of G-Hes application in DN by providing novel findings and overall information on the targets of G-Hes.

## 2. Materials and Methods

### 2.1. Animals

All mice were housed in a temperature-controlled (24 ± 2 °C) room with a 12 h/12 h light/dark cycle. The mice were fed food and water ad libitum with a standard diet (Oriental Yeast, Tokyo, Japan) containing 59% kcal of carbohydrates, 14% kcal of fat, and 26% kcal of protein. The Hiroshima University Animal Care and Use Committee approved all animal experiments (Permit Number: C22-53). Animal studies were performed in accordance with the protocols approved by the Hiroshima University Animal Care and Use Committee from 1 July to 25 December 2022.

### 2.2. Diabetic Nephropathy Mouse Model

The streptozotocin (STZ)-induced DN model was developed using seven-week-old male CD-1 (ICR) mice (Charles River Japan, Hino, Japan). Mice were divided into two groups based on body weight: the control (*n* = 5) and STZ groups (*n* = 15). After 10 h of fasting, diabetes was induced via intraperitoneal (*i.p.*) injections of STZ (Nacalai Tesque, Osaka, Japan) at a dose of 100 mg/kg twice at an interval of one day. Blood glucose levels were measured two days after the second STZ administration, and the STZ group was divided into two groups based on the blood glucose levels: DM group, *n* = 8; G-Hes group, *n* = 7. These groups were administered distilled water and distilled water containing 1% G-Hes (Hayashibara, Okayama, Japan) for 4 weeks, respectively. The body weight and fasting blood glucose levels were measured weekly. Water intake was measured every two days. The average daily G-Hes intake was 239 mg per mouse. The kidneys were removed after cervical dislocation at autopsy and used for each analysis after four weeks of STZ administration.

### 2.3. Dosage Information

The STZ group was administered distilled water containing 1% G-Hes for 4 weeks.

### 2.4. DNA Microarray Analysis

DNA microarray analysis was performed as previously described [20]. Briefly, total RNA was extracted from the renal tissues of the three groups using the RNeasy Lipid Tissue Kit (Qiagen Sciences, Germantown, MD, USA). The RNA samples were used for DNA microarray analysis (44 K whole-mouse genome microarray; Agilent Technologies, Palo Alto, CA, USA). Agilent Feature Extraction (version 9.5) (Santa Clara, CA, USA) was used for statistical analysis of the gene expression data.

### 2.5. Gene Functional Analysis

Gene Ontology (GO) enrichment analysis was used for efficient interpretation to organize functionally related genes into biological processes, molecular functions, and cellular components, facilitating the organization of functionally related genes into biologically meaningful modules [21]. The 20 highest modules with fold enrichment > 1.90 were selected. We ensured that the analysis included no duplication of the official gene symbols, ensuring accuracy and reliability.

### 2.6. Quantitative Polymerase Chain Reaction (PCR) Analysis

Total RNA was isolated using an RNeasy Lipid Tissue Kit (Qiagen Sciences, Germantown, MD, USA). The reverse transcriptase reaction was performed with total RNA as a template to synthesize the cDNA using the ReverTra Ace qPCR RT Kit (TOYOBO, Osaka, Japan). The cDNA was used for quantitative PCR analysis with the THUNDERBIRD SYBR qPCR Mix (TOYOBO) and StepOnePlus (Applied Biosystems, Foster City, CA, USA). The primers used for PCR analyses were as follows: Krt20, F, 5′-TCACCGAAGTCTGAGTTCCTC-3′, and R, 5′-CTCATTACGGCTTTGGAGACAG-3′; Kim1, F, 5′-ACATATCGTGGAATCACAACGAC-3′, and R, 5′-ACTGCTCTTCTGATAGGTGACA-3′; TNF-α, F, 5′-CCGATGGGTTGTACCTTGTC-3′, and R, 5′-CGGACTCCGCAAAGTCTAAG-3′; EMR1, F, 5′-ATTGTGGAAGCATCCGAGAC-3′, and R, 5′-GTAGGAATCCCGCAATGATG-3′; CCL2, F, 5′-GGTCCCTGTCATGCTTCTGG-3′, and R, 5′-CCTTCTTGGGGTCAGCACAG-3′; TGFβ, F, 5’-GGCACCATCCATGACATGAA-3,’ and R, 5’-TTCTCTGTGGAGCTGAAGCAAT-3′; α-smooth muscle actin (αSMA), F, 5′-GGCTCTGGGCTCTGTA-3′, and R, 5′-CTCTTGCTCTGGGCTTCATC-3′; Col1, F, 5′-CCCAAGGAAAAGAAGC-3′, and R, 5′-ACATTAGGCGCAGGAAGGTCA-3′; fibronectin1, F, 5′-GGTGACACTTATGAGCGCCCTAAA-3′, and R, 5′-AACATGTAACCACCAGTCTCATGTG-3′; L19, F, 5′-GGCATAGGGAAGAGGAAGG-3′, and R, 5′-GGATGTGCTCCATGAGGATGC-3′.

### 2.7. Histological Analysis

The renal tissues were fixed in 10% formalin solution at room temperature (RT, approximately 25  ±  5 °C) and embedded in paraffin, and 5 mm-thick sections were prepared. Subsequently, the sections were processed for hematoxylin and eosin staining to determine the overall histology and Azan–Mallory staining to examine the renal fibrosis degree [19]. For immunohistochemical analysis, the renal tissues were fixed in 4% paraformaldehyde at RT, embedded in paraffin, and 5 mm-thick sections were prepared. The sections were subsequently incubated with a solution consisting of 2% bovine serum albumin, 0.1% sodium azide, 5% fetal bovine serum, 5% normal goat serum, and 0.2% Triton-X-100 for 1 h at RT. The sections were then incubated with primary antibody (rabbit anti-C3 (1:500; Proteintech, Rosemont, IL, USA) and biotinylated goat anti-rabbit IgG (H+L) (Vector Laboratories, Newark, CA, USA), followed by incubation for 5 min at RT with the avidin–biotin peroxidase complex (Vector Laboratories). All images were captured using an Olympus BX53 microscope (Olympus, Tokyo, Japan) and analyzed using Nikon Elements imaging (Nikon, Tokyo, Japan).

### 2.8. Cell Culture and Western Blot Analysis

The human proximal tubular cell line HK-2 was cultured in Dulbecco’s modified medium with low glucose with 10% fetal bovine serum, 100 units/mL penicillin, and 100 μg/mL streptomycin in 5% CO_2_ at 37 °C in a humidity incubator. For experimental purposes, the cells were exposed to 20 ng/mL of TGF-β1 (R&D Systems, Minneapolis, MN, USA) for 48 h in the presence of hesperetin (Tokyo Chemical Industry Co., Tokyo, Japan) at 30 μM. Cell lysate was prepared with RIPA buffer containing 1 mM PMSF, 20 µg/mL aprotinin, and phosphatase inhibitor cocktail (Nacalai Tesque, Osaka, Japan). Protein concentration was quantified using the DC Protein Assay (Bio-Rad Laboratories, Inc. Hercules, CA, USA). A total of 15 µg of protein for each sample was loaded into the wells of the SDS-PAGE gel, run for 100 min at 28 mA, and transferred to PVDF membranes. The membranes were blocked in 4% skim milk in PBS for 1 h, then incubated in the primary antibody overnight at 4 °C for anti-E-cadherin (1:1000, Cell Signaling, Danvers, MA, USA, 24E10), anti-N-cadherin (1:1000, Cell Signaling, D4R1H), anti-snail (1:1000, Cell Signaling, Danvers, MA, USA, C15D3), anti-ZEB1 (1:1000, Cell Signaling, Danvers, MA, USA, D80D3), and anti-β-Actin (1:5000, FUJIFILM Wako, Tokyo, Japan, 010-27841). Membranes were further incubated with the respective secondary antibodies (Cell Signaling). Can Get Signal™ Immunoreaction Enhancer Solution (TOYOBO) was used for the antibody reaction. Signals were detected using Western Lighting-ECL (PerkinElmer, Inc. Shelton, CT, USA).

### 2.9. Statistical Analysis

Data are presented as the means ± standard error. Differences in the significance levels between the compared groups were determined using the Student’s *t*-test or one-way analysis of variance, followed by Dunnett’s test. Statistical analyses were conducted using GraphPad Prism 7 software, with *p*-values < 0.05 considered statistically significant, as denoted in the figure legends.

## 3. Results

We used a mouse model of diabetes induced by STZ administration to investigate the potential effects of G-Hes on DN. ICR mice were randomly divided into three groups: control, diabetes mellitus (DM), and G-Hes. In the DM and G-Hes groups, mice were *i.p.* injected with 100 mg/kg STZ twice at an interval of one day. The final fasting blood glucose levels were significantly elevated in this model (more than 400 mg/dL). The G-Hes group was administered G-Hes (1% *w*/*v*) in drinking water 2 d after initiating STZ administration. We measured the body weight and fasting blood glucose levels over 4 weeks consecutively, and excised the kidneys for further analysis.

### 3.1. G-Hes Supplementation Suppressed an Increased Kidney Weight Without Any Change in Blood Glucose Level

**Figure 1:**
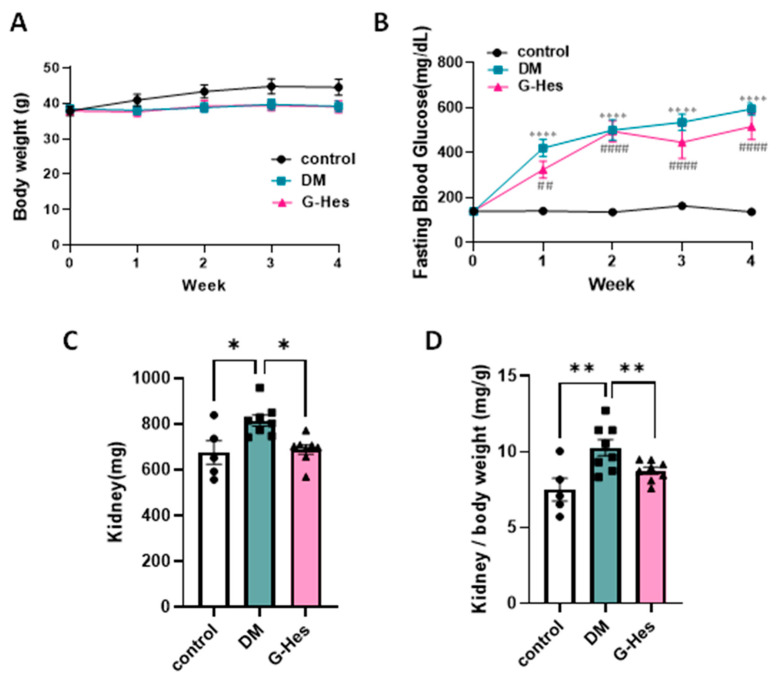
The preventive effect of G-Hes supplementation on an increased kidney weight without any change in the blood glucose level. (**A**,**B**) Body weight and blood glucose level showed no difference between the DM group (*n* = 8) and G-Hes group (*n* = 7). (**C**,**D**) Kidney weight and relative kidney weight of the G-Hes group were lower than the DM group. All values are expressed as the means ± S.E. * *p* < 0.05, ** *p* and ## *p* < 0.01, **** *p* and #### *p* < 0.0001 as determined by the Student’s *t*-test (control, *n* = 5; DM, *n* = 8; G-Hes, *n* = 7).

The DM group revealed lower body weight and higher fasting blood glucose levels than the control group; however, differences in the body weight or fasting blood glucose levels between the DM and G-Hes groups were absent (Figure 1A,B). G-Hes supplementation substantially suppressed the increase in kidney weight during DN development (Figure 1C,D), suggesting the preventive effect of G-Hes but not through a mechanism of blood glucose control.

### 3.2. G-Hes Supplementation Downregulated the mRNA Expressions Related to Renal Tubule Injury, Fibrosis, and Immune Responses

**Figure 2:**
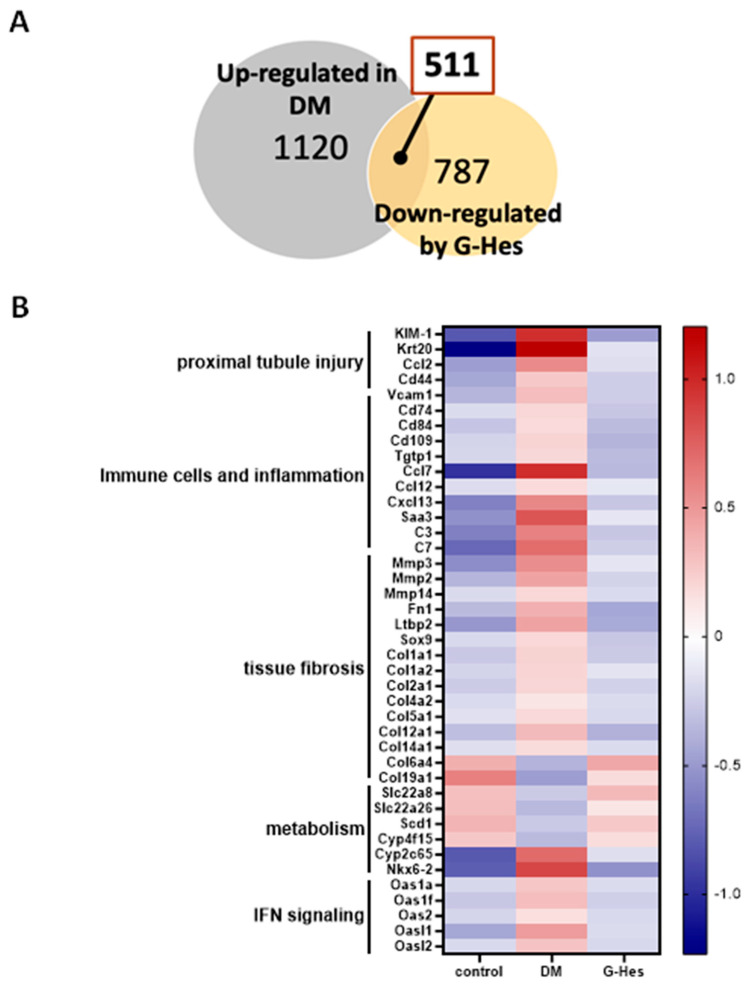
Molecular cues for the preventive effect of G-Hes supplementation. (**A**) Venn diagram shows the renal genes that were upregulated in the DM group and downregulated by G-Hes supplementation. (**B**) Selected gene expression related to renal pathologies by hyperglycemia.

We conducted DNA microarray analysis to examine the renal expression patterns across all genes to explore the mechanism underlying the protective effect of G-Hes. Numerous genes were deemed differentially expressed between the control, DM, and G-Hes groups if *p* < 0.05 with a ≥1.5 or ≤0.67-fold change. Based on the DNA microarray analysis, of the 1120 genes upregulated during DM development compared with the control group, G-Hes supplementation decreased the expression of 511 genes compared with the DM group (Figure 2A). This group of 511 genes included the renal tubular injury (Kim1, Krt20, Vcam1, and Ccl2) and tissue fibrosis marker genes (fibronectin 1 (Fn1), latent transforming growth factor beta-binding protein 2 (Ltbp2), Sox9, and Collagen1a1) (Figure 2B).

**Figure 3:**
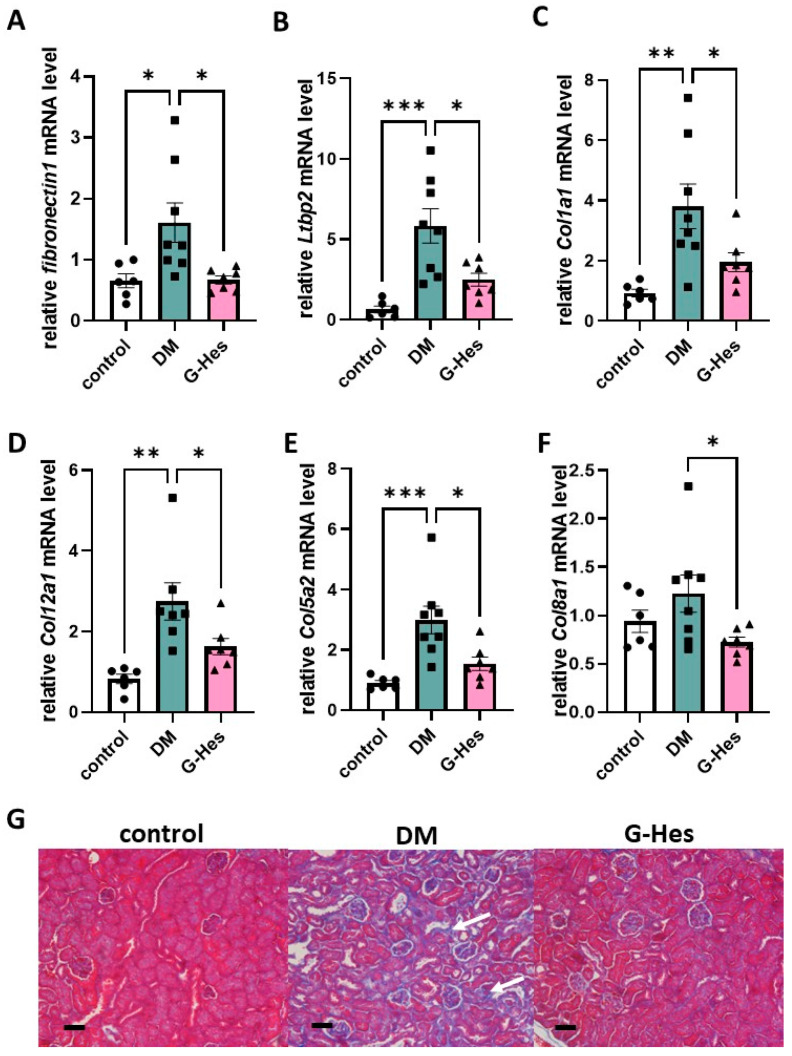
The preventive effect of G-Hes supplementation on renal fibrosis. (**A**–**F**) Relative mRNA expression of renal fibrosis-related genes in three groups. (**G**) Kidney tissues were isolated and paraffin-embedded sections were subjected to Azan-Mallory (AZM) staining. Tubulointerstitial fibrosis in the DM group (the white arrow). Values are the means ± SEM. Statistical analysis was performed with the Student’s *t*-test. * *p* < 0.05; ** *p* < 0.01; *** *p* < 0.001 (control, *n* = 5; DM, *n* = 8; G-Hes, *n* = 7). Scale bars represent 100 μm.

These results suggest that G-Hes supplementation suppresses mRNA expression of the tissue fibrosis-related genes upregulated by DN development. Quantitative PCR analysis revealed that G-Hes treatment downregulated the mRNA expression of Fn1, Ltbp2, and members of the collagen family (Figure 3A–F). Paraffin-embedded kidney sections were subjected to Azan–Mallory staining, which revealed that G-Hes supplementation suppressed tubular dilation, atrophy, and tubulointerstitial fibrosis, which were observed in the DM group (Figure 3G).

### 3.3. G-Hes Supplementation Suppressed Activation of the Complement System and Glomerular Hypertrophy

**Figure 4:**
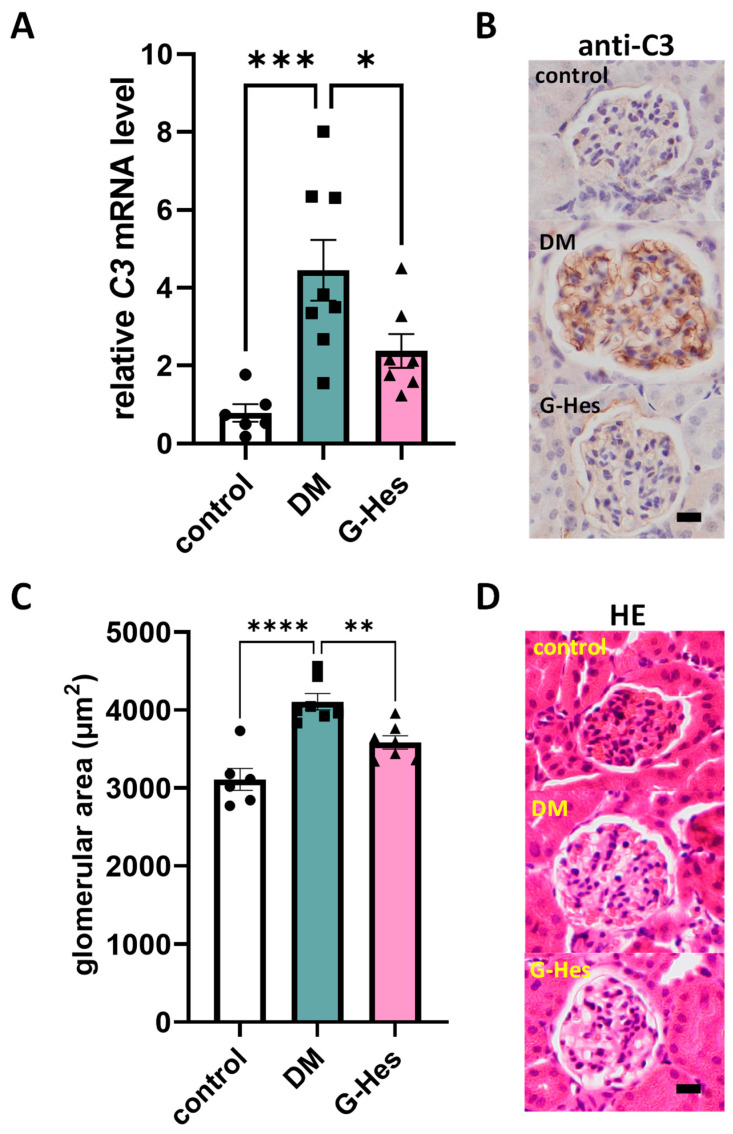
The preventive effect of G-Hes supplementation on C3 protein accumulation and the glomerular area. (**A**) Relative mRNA expression of the renal C3 gene in three groups. (**B**) Immunohistochemical localization of the C3 protein in the mouse kidney. Kidney tissues of three groups were isolated, and paraffin-embedded sections were subjected to anti-C3 staining. (**C**,**D**) Quantification of the average glomerular cross-sectional area. Representative image of the kidney histological staining with H&E reagents. Values are the means ± SEM. Statistical analysis was performed with the Student’s *t*-test. * *p* < 0.05; ** *p* < 0.01; *** *p* < 0.005; **** *p* < 0.001 (control, *n* = 5; DM, *n* = 8; G-Hes, *n* = 7). Scale bars represent 20 μm.

Of the 511 genes, G-Hes treatment downregulated mRNA expression of the complement component C3 mRNA expression. This study focused on the effect of G-Hes supplementation on the complement system, as examined by the C3 expression levels in the kidneys of the STZ-treated mice. We further performed immunohistochemical analyses of the renal sections of the STZ-treated mice for 4 weeks because G-Hes treatment decreased the renal C3 mRNA expressions (Figure 4A). We found that glomerular C3 protein accumulation suppressed G-Hes supplementation (Figure 4B), suggesting that G-Hes exhibits a renoprotective effect through the suppression of complement system activation caused by hyperglycemia. Subsequently, we measured the glomerular area as a glomerular injury indicator in the renal sections of the three groups. G-Hes supplementation substantially suppressed glomerular hypertrophy observed during DM development (Figure 4C,D). Glomerular hypertrophy was mainly related to hyperfiltration and the increased mesangial matrix, indicating that G-Hes improves mesangial cell function.

### 3.4. G-Hes Supplementation Suppressed Mesangial Expansion and Suppressed the Increase in IFN-γ-Related Gene Expression

**Figure 5:**
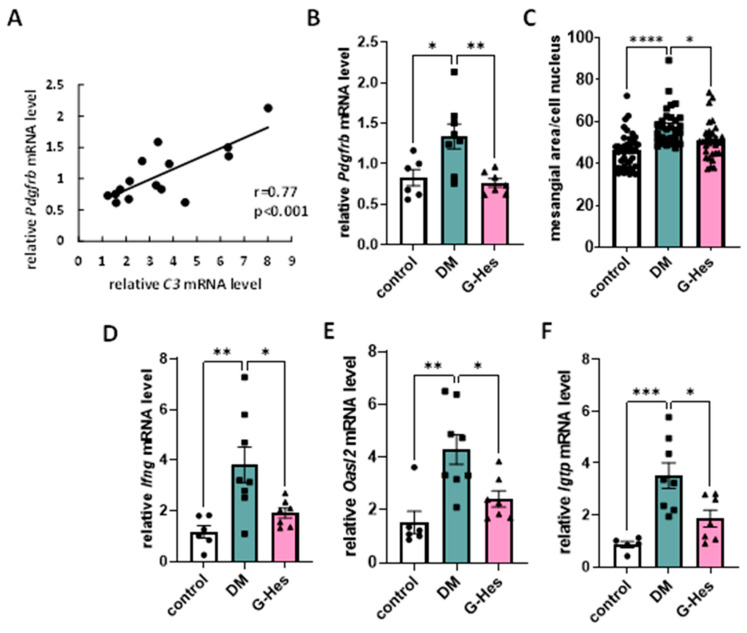
Response of renal mRNA expressions to G-Hes supplementation. (**A**) Total RNA from the DMA and G-Hes groups (*n* = 15) was subjected to quantitative PCR to examine the renal mRNA expression level of the *C3* and *PDGFRβ* genes. The relative mRNA expression level of each gene was analyzed with the Pearson’s correlation coefficient. (**B**) The relative mRNA expression level of PDGFRβ was determined by quantitative PCR. (**C**) Kidney tissues of the three groups were isolated, and paraffin-embedded sections were subjected to anti-αSMA staining to quantify the average mesangial cell area in the glomerulus. (**D**–**F**) Relative mRNA expression of the renal IFN signaling-related genes in the three groups. All values are expressed as the means ± S.E. * *p* < 0.05; ** *p* < 0.01; *** *p* < 0.005; **** *p* < 0.001 (control, *n* = 5; DM, *n* = 8; G-Hes, *n* = 7).

We investigated the pathological relationship between C3 and the expression of other genes to characterize C3 expression during DN progression [22]. Interestingly, C3 mRNA expression was positively correlated with the mRNA expression of the platelet-derived growth factor receptor-β (PDGFR-β) (Figure 5A), which is considered a mediator of mesangial injury. Additionally, G-Hes supplementation decreased the mRNA expression of PDGFR-β (Figure 5B). Furthermore, G-Hes substantially decreased the αSMA-positive mesangial area (Figure 5C), indicating that G-Hes supplementation suppressed the mesangial expansion during DN progression. Furthermore, the 511 genes identified by the DNA microarray analyses included genes such as the interferon-gamma (IFN-γ) gene involved in innate immune responses. Particularly, several genes of the 2′-5′ oligoadenylate synthase (OAS) family (Oas1, Oas2, Oas3, OAS-like protein 1 (Oasl1), and Oasl2), called IFN-stimulated genes, were identified as being downregulated by G-Hes. mRNA expression of IFN-γ-stimulated genes such as Oasl2 and IFN-γ-induced GTPase were increased in the DM group, whereas G-Hes supplementation suppressed the expression of these genes (Figure 5D–F). IFN is recognized as a crucial factor in the renal pathologies of DN, suggesting a functional relationship between IFN signaling and the renoprotective effect of G-Hes.

### 3.5. Hesperetin Alleviates Epithelial–Mesenchymal Transition by TGF-β in Renal Epithelial Cells

**Figure 6:**
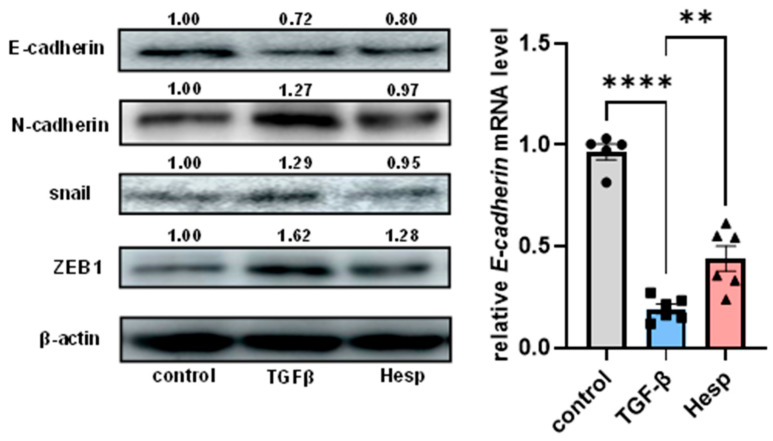
Hesperetin inhibits EMT in TGF-β-treated HK-2 cells. Protein expressions of E-cadherin, N-cadherin, snail, and ZEB1 were assessed after 48 h of treatment with TGF-β and hesperetin (Hesp, 30 μM). E-cadherin mRNA expression was examined after 48 h of treatment with TGF-β and hesperetin (Hesp). Error bars indicate the S.E.; ** *p* < 0.01, **** *p* < 0.001, compared to the control group. *n* = 6.

During DN development, epithelial–mesenchymal transition (EMT) is considered as a pathological process in the renal tubular cells, which is closely related to extracellular matrix accumulation. In this study, we examined the effect of hesperetin on EMT in the human proximal tubular cell line HK-2, because the absorption of G-Hes is associated with the cleavage of the sugar moiety under the action of hydrolyzing enzymes in the gastrointestinal tract. Hesperetin supplementation inhibited the expression of EMT markers such as N-cadherin in TGF-β-treated HK-2 cells (Figure 6), whereas the decreased E-cadherin expression in response to TGF-β was partially abolished by the hesperetin treatment (Figure 6), suggesting a possible effect of G-Hes supplementation on the renal tubular EMT in vivo.

## 4. Discussion

Polyphenol supplementation has increasingly been considered as an effective approach to prevent DN, the primary cause of end-stage renal failure [8,9,10,11,12]. Although polyphenols exert protective effects against DN through their hypoglycemic effects, their direct renoprotective effects have not been completely investigated. This study revealed that G-Hes supplementation prevented renal immune activation and tubulointerstitial fibrosis without considerable alternations in the blood glucose levels. On the basis of DNA microarray analysis, G-Hes supplementation suppressed the increase in the mRNA expression of the markers of proximal tubule injury and renal complement factors that correlated with DN severity, accompanied by a decrease in glomerular and mesangial areas.

Renal tubular epithelial cells, an intrinsic cell type present outside the renal tubules, are responsible for urinary glucose reabsorption. Renal tubulointerstitial injury is an important pathological basis for DN progressing to end-stage renal disease in humans. Hyperglycemia and activation of the renin-angiotensin system are associated with oxidative stress, hypoxia, and endoplasmic reticulum stress, consequently triggering apoptosis of the renal tubular cells and epithelial–mesenchymal transition (EMT) during renal tubulointerstitial injury [23]. Interestingly, the sodium-glucose cotransporter 2 inhibitors, a new class of hypoglycemic drugs, have previously been found to ameliorate kidney structure and decelerate DN progression, possibly through their protective effect on injury of the proximal renal tubular cells [24]. Recent studies on drug-induced nephrotoxicity have identified renal genes including the *keratin* (*Krt*) gene family as indicators of proximal tubule cell injury and proliferation [25]. Herein, DNA microarray analysis demonstrated decreased mRNA expression of the proximal tubule injury markers (*Kim1*, *Krt20*, *Vcam1*, *Ccl2*, and *Cd44*), suggesting that G-Hes supplementation protected the proximal tubules. Several studies have demonstrated that dietary hesperidin decreases renal Kim1 expression in acetaminophen- or trichloroethylene-induced kidney disease in mice [26,27]. Recent studies proposed urinary flavanone concentrations as biomarkers of dietary flavanone intakes [28]. In fact, we have previously measured the hesperetin concentration (45.1 ± 15.3 µM) in urine of a different DM mouse model with a combination of low-dose STZ injection and a high-fat diet fed with 1% G-Hes, suggesting that hesperetin in urine directly alleviates proximal tubule injury. Hesperetin has been reported to attenuate oxidative stress-induced apoptosis in human renal proximal tubular epithelial HK-2 cells treated with cisplatin by activating the Nrf2 signaling pathway [29]. Interestingly, G-Hes supplementation suppressed renal tubulointerstitial injury, possibly by reducing oxidative stress because hesperetin inhibited xanthine oxidase activity in vitro and in vivo [19]. During DN development, apoptosis and EMT are the major cell remodeling processes in the renal tubular cells, which are closely linked to increased extracellular matrix and interstitial fibrosis [30]. Recently, hesperetin has been reported to inhibit EMT in TGF-β-treated podocytes by regulation of the mTOR pathway [31]. Additionally, other studies have revealed that hesperidin suppresses EMT in the lung and gastric tissues [32,33]. Although further studies are needed to examine renal oxidative stress markers, this study suggests an inhibitory effect of G-Hes supplementation on the renal tubular EMT through reducing oxidative stress in vivo.

Recently, increasing evidence has indicated a pathological role of the complement system in DN progression [34]. The complement component C3 plays a key role in activating the complement system in both the classical and alternative pathways. In humans, C3 glomerulopathy is characterized by C3 deposits and the dysregulation of alternative complement pathways. Pathologies based on glomerular complement activation are the primary drivers of kidney disease. In previous studies on STZ-treated diabetic mice, glomerular complement component 3a (C3a) receptor 1 (C3aR) expression was observed in the early phase of DN, suggesting the pathological role of C3a in DN development [35]. In the STZ-treated diabetic mice, C3a inhibition using a C3aR antagonist alleviated albumin excretion and glomerular injury [36]. Resveratrol has previously been shown to attenuate proteinuria and other pathological characteristics in idiopathic membranous nephropathy model mice, partially through direct anticomplement activity [37]. Additionally, this study revealed that G-Hes supplementation reduced renal C3 mRNA expression and glomerular C3-positive areas. This suggests that the inhibitory effect of G-Hes on C3 expression is associated with decreased glomerular hypertrophy. IFN-γ has previously been shown to increase *C3* gene expression in glomerular epithelial cells in a time- and dose-dependent manner [38]. IFN reportedly induces the expression of numerous IFN-stimulated genes that act as IFN-inducible antiviral effectors [39]. Among the IFN-stimulated genes, Oas responds to exogenous nucleic acids and exerts antiviral effects. Interestingly, G-Hes supplementation in this study suppressed mRNA expression of the *IFN-γ* gene and genes of the Oas family, such as *Oas1*, *Oas2*, *Oas3*, *Oasl1*, and *Oasl2*, suggesting that a decrease in the IFN-γ level is a crucial event in regulating C3 mRNA expression for the preventive effect of G-Hes on glomerular inflammation and hypertrophy. Although the pathological role of IFN-γ in the kidney in type II diabetes has been discussed to date, further studies are required to clarify the IFN-γ-related gene expression to elucidate the mechanism of the preventive effect of G-Hes supplementation. Interestingly, C3 mRNA expression was positively correlated with the PDGFR-β mRNA expression. Furthermore, a transgenic mouse with constitutive PDGFR-β activation, particularly in the renal mesenchymal cells, was recently used to demonstrate the pathological role of PDGF signaling for glomerular mesangial proliferation and interstitial fibrosis [40]. Taken together with the observation that G-Hes supplementation suppressed PDGFR-β expression and mesangial expansion, further experiments are warranted to demonstrate the pathological relationship between glomerular C3 expression and PDGF signaling to understand the mechanism underlying the effect of dietary G-Hes.

This study demonstrated that G-Hes supplementation prevents renal immune activation and tubulointerstitial fibrosis and that its renoprotective effect is possibly due to the amelioration of renal tubule injury, excessive activation of the innate immune system including the complement system, and expansion of the mesangial matrix. This study provides novel insights into the dietary effects of polyphenols on kidney damage through their hypoglycemic effects, and sheds light on the complement system’s pathological role, thus proposing a new strategy to treat DN in the development of functional food products.

## 5. Limitations of the Study

STZ is widely used to study diabetic complications. Due to the nonspecific cytotoxicity of high-dose STZ, an alternative diabetic model using a combination of low-dose STZ and a high-fat diet was established. Further investigations are required to determine whether the present findings can be reproduced in the diabetic mouse model. A detailed explanation on the molecular mechanism of the preventive effect of G-Hes is needed before human trials.

## Data Availability

All data are available from the corresponding authors upon reasonable request.

## References

[1] Bikbov B., Purcell C.A., Levey A.S., Smith M., Abdoli A., Abebe M., Adebayo O.M., Afarideh M., Agarwal S.K., Agudelo-Botero M. (2020). Global, regional, and national burden of chronic kidney disease, 1990–2017: A systematic analysis for the Global Burden of Disease Study 2017. Lancet.

[2] Tang S.C., Yiu W.H. (2020). Innate immunity in diabetic kidney disease. Nat. Rev. Nephrol..

[3] Yang M., Zhang C. (2023). The role of innate immunity in diabetic nephropathy and their therapeutic consequences. J. Pharm. Anal..

[4] Najafian B., Alpers C.E., Fogo A.B. (2011). Pathology of human diabetic nephropathy. Contrib. Nephrol..

[5] Maezawa Y., Takemoto M., Yokote K. (2015). Cell biology of diabetic nephropathy: Roles of endothelial cells, tubulointerstitial cells and podocytes. J. Diabetes Investig..

[6] Thipsawat S. (2021). Early detection of diabetic nephropathy in patient with type 2 diabetes mellitus: A review of the literature. Diab. Vasc. Dis. Res..

[7] Hu Q., Chen Y., Deng X., Li Y., Ma X., Zeng J., Zhao Y. (2023). Diabetic nephropathy: Focusing on pathological signals, clinical treatment, and dietary regulation. Biomed. Pharmacother..

[8] Jin Q., Liu T., Qiao Y., Liu D., Yang L., Mao H., Ma F., Wang Y., Peng L., Zhan Y. (2023). Oxidative stress and inflammation in diabetic nephropathy: Role of polyphenols. Front. Immunol..

[9] Huang K., Huang J., Xie X., Wang S., Chen C., Shen X., Liu P., Huang H. (2013). Sirt1 resists advanced glycation end products-induced expressions of fibronectin and TGF-β1 by activating the Nrf2/ARE pathway in glomerular mesangial cells. Free Radic. Biol. Med..

[10] Chang C.C., Chang C.Y., Wu Y.T., Huang J.P., Yen T.H., Hung L.M. (2011). Resveratrol retards progression of diabetic nephropathy through modulations of oxidative stress, proinflammatory cytokines, and AMP-activated protein kinase. J. Biomed. Sci..

[11] Gomes I.B., Porto M.L., Santos M.C.L., Campagnaro B.P., Pereira T.M., Meyrelles S.S., Vasquez E.C. (2014). Renoprotective, anti-oxidative and anti-apoptotic effects of oral low-dose quercetin in the C57BL/6J model of diabetic nephropathy. Lipids Health Dis..

[12] Wang C., Pan Y., Zhang Q.Y., Wang F.M., Kong L.D. (2012). Quercetin and allopurinol ameliorate kidney injury in STZ-treated rats with regulation of renal NLRP3 inflammasome activation and lipid accumulation. PLoS ONE.

[13] Galati E.M., Monforte M.T., Kirjavainen S., Forestieri A.M., Trovato A., Tripodo M.M. (1994). Biological effects of hesperidin, a citrus flavonoid. (Note I): Antiinflammatory and analgesic activity. Farmaco.

[14] Monforte M.T., Trovato A., Kirjavainen S., Forestieri A.M., Galati E.M., RB L.C. (1995). Biological effects of hesperidin, a Citrus flavonoid. (note II): Hypolipidemic activity on experimental hypercholesterolemia in rat. Farmaco.

[15] Galati E.M., Trovato A., Kirjavainen S., Forestieri A.M., Rossitto A., Monforte M.T. (1996). Biological effects of hesperidin, a Citrus flavonoid. (Note III): Antihypertensive and diuretic activity in rat. Farmaco.

[16] Pyrzynska K. (2022). Hesperidin: A review on extraction methods, stability and biological activities. Nutrients.

[17] Hijiya H., Miyake T. (1991). Alpha-glycosyl hesperidin, and its preparation and uses. Eur. Pat. Publ..

[18] Yamada M., Tanabe F., Arai N., Mitsuzumi H., Miwa Y., Kubota M., Chaen H., Kibata M. (2006). Bioavailability of glucosyl hesperidin in rats. Biosci. Biotechnol. Biochem..

[19] Kumrungsee T., Kariya T., Hashimoto K., Koyano T., Yazawa N., Hashimoto T., Sanada Y., Matsuyama M., Sotomaru Y., Sakurai S. (2019). The serum amyloid A3 promoter-driven luciferase reporter mice is a valuable tool to image early renal fibrosis development and shows the therapeutic effect of glucosyl-hesperidin treatment. Sci. Rep..

[20] Okazaki Y., Ohshima N., Yoshizawa I., Kamei Y., Mariggiò S., Okamoto K., Maeda M., Nogusa Y., Fujioka Y., Izumi T. (2010). A novel glycerophosphodiester phosphodiesterase, GDE5, controls skeletal muscle development via a non-enzymatic mechanism. J. Biol. Chem..

[21] Aleksander S.A., Balhoff J., Carbon S., Cherry J.M., Drabkin H.J., Ebert D., Feuermann M., Gaudet P., Harris N.L., Hill D.P. (2023). The gene ontology knowledgebase in 2023. Genetics.

[22] Petr V., Thurman J.M. (2023). The role of complement in kidney disease. Nat. Rev. Nephrol..

[23] Liu Z., Nan P., Gong Y., Tian L., Zheng Y., Wu Z. (2023). Endoplasmic reticulum stress-triggered ferroptosis via the XBP1-Hrd1-Nrf2 pathway induces EMT progression in diabetic nephropathy. Biomed. Pharmacother..

[24] Vallon V., Verma S. (2021). Effects of SGLT2 inhibitors on kidney and cardiovascular function. Annu. Rev. Physiol..

[25] Ma Z., Hu X., Ding H.F., Zhang M., Huo Y., Dong Z. (2022). Single-nucleus transcriptional profiling of chronic kidney disease after cisplatin nephrotoxicity. Am. J. Pathol..

[26] Ahmad S.T., Arjumand W., Nafees S., Seth A., Ali N., Rashid S., Sultana S. (2012). Hesperidin alleviates acetaminophen induced toxicity in Wistar rats by abrogation of oxidative stress, apoptosis and inflammation. Toxicol. Lett..

[27] Siddiqi A., Nafees S., Rashid S., Sultana S., Saidullah B. (2015). Hesperidin ameliorates trichloroethylene-induced nephrotoxicity by abrogation of oxidative stress and apoptosis in wistar rats. Mol. Cell Biochem..

[28] Tahiri I., Garro-Aguilar Y., Cayssials V., Achaintre D., Mancini F.R., Mahamat-Saleh Y., Boutron-Ruault M.C., Kühn T., Katzke V., Boeing H. (2020). Urinary flavanone concentrations as biomarkers of dietary flavanone intakes in the European Prospective Investigation into Cancer and Nutrition (EPIC) study. Br. J. Nutr..

[29] Chen X., Wei W., Li Y., Huang J., Ci X. (2019). Hesperetin relieves cisplatin-induced acute kidney injury by mitigating oxidative stress, inflammation and apoptosis. Chem. Biol. Interact..

[30] Tang G., Li S., Zhang C., Chen H., Wang N., Feng Y. (2021). Clinical efficacies, underlying mechanisms and molecular targets of Chinese medicines for diabetic nephropathy treatment and management. Acta Pharm. Sin. B.

[31] Choi D., Kim C.L., Kim J.E., Mo J.S., Jeong H.S. (2020). Hesperetin inhibit EMT in TGF-β treated podocyte by regulation of mTOR pathway. Biochem. Biophys. Res. Commun..

[32] Liang Z., Zhang Y., Xu Y., Zhang X., Wang Y. (2022). Hesperidin inhibits tobacco smoke-induced pulmonary cell proliferation and EMT in mouse lung tissues via the p38 signaling pathway. Oncol. Lett..

[33] Liang Z., Song J., Xu Y., Zhang X., Zhang Y., Qian H. (2022). Hesperidin reversed long-term N-methyl-N-nitro-N-nitroguanidine exposure induced EMT and cell proliferation by activating autophagy in gastric tissues of rats. Nutrients..

[34] Flyvbjerg A. (2017). The role of the complement system in diabetic nephropathy. Nat. Rev. Nephrol..

[35] Morigi M., Perico L., Corna D., Locatelli M., Cassis P., Carminati C.E., Bolognini S., Zoja C., Remuzzi G., Benigni A. (2020). C3a receptor blockade protects podocytes from injury in diabetic nephropathy. JCI Insight..

[36] Angeletti A., Cantarelli C., Petrosyan A., Andrighetto S., Budge K., D’Agati V.D., Hartzell S., Malvi D., Donadei C., Thurman J.M. (2020). Loss of decay-accelerating factor triggers podocyte injury and glomerulosclerosis. J. Exp. Med..

[37] Wu C.C., Huang Y.S., Chen J.S., Huang C.F., Su S.L., Lu K.C., Lin Y.F., Chu P., Lin S.H., Sytwu H.K. (2015). Resveratrol ameliorates renal damage, increases expression of heme oxygenase-1, and has anti-complement, anti-oxidative, and anti-apoptotic effects in a murine model of membranous nephropathy. PLoS ONE.

[38] Sacks S.H., Zhou W., Pani A., Campbell R.D., Martin J. (1993). Complement C3 gene expression and regulation in human glomerular epithelial cells. Immunology.

[39] Choi U.Y., Kang J.S., Hwang Y.S., Kim Y.J. (2015). Oligoadenylate synthase-like (OASL) proteins: Dual functions and associations with diseases. Exp. Mol. Med..

[40] Buhl E.M., Djudjaj S., Klinkhammer B.M., Ermert K., Puelles V.G., Lindenmeyer M.T., Cohen C.D., He C., Borkham-Kamphorst E., Weiskirchen R. (2020). Dysregulated mesenchymal PDGFR-β drives kidney fibrosis. EMBO Mol. Med..

